# DNA methylation informs adult cardiovascular risk and prevention for low birthweight individuals

**DOI:** 10.7189/jogh.16.04242

**Published:** 2026-08-14

**Authors:** John Yen Tang, Nicole Ying Ting Ng, Alice Wing Sze Kwok, Christopher Chun Yu Mak, Yiu-Fai Cheung, Brian Hon Yin Chung

**Affiliations:** Department of Paediatrics and Adolescent Medicine, School of Clinical Medicine, Li Ka Shing Faculty of Medicine, The University of Hong Kong, Hong Kong SAR, China

**Keywords:** Barker’s hypothesis, cardiovascular disease risk, DNA methylation, epigenetics, Mendelian randomisation

## Abstract

**Background:**

Barker’s hypothesis posits that adverse *in utero* exposures, often reflected by low birthweight, increase adult cardiovascular disease (CVD) risk. However, underlying mechanisms remain unclear; identifying potentially causal mediators is crucial for developing effective interventions. Emerging research suggests epigenetic modifications may influence CVD traits from early life. We aimed to identify DNA methylation (DNAm) signatures that mediate birthweight and CVD traits and to establish their biological relevance across three developmental time points using multi-omics integration.

**Methods:**

We identified DNAm mediators using epigenetic Mendelian randomisation (MR). The exposures were 261 birthweight-associated CpG sites (BW-CpGs) from epigenome-wide association studies, instrumented by *cis*-methylation quantitative trait loci (*cis*-mQTLs) in European-ancestry participants from the Accessible Resource for Integrative Epigenomic Studies measured at three developmental time points: birth (mean gestational age = 40 weeks), childhood (mean age = 7.49 years), and adolescence (mean age = 17.14 years). The outcomes included adult cardiovascular events and lipid traits from genome-wide association studies (n = 98,048–547,261). Sensitivity analyses included colocalisation, Steiger’s test, Cochran’s Q, and pleiotropy-robust methods. We interrogated DNAm persisting across development using omics datasets.

**Results:**

Using *cis*-mQTLs measured at birth, we found 230 associations between 216 BW-CpGs and CVD traits, including coronary artery disease, hypertension, stroke, cholesterol, and triglycerides (false discovery rate-corrected *P* < 0.05), with effect size directions consistent with Barker’s hypothesis. A total of 42 associations were robust following sensitivity analyses. Persistent DNAm changes across development were observed at *ASGR1* and *KDM2B*. Multi-omics interrogation prioritised a promising therapeutic candidate (*ASGR1*).

**Conclusions:**

Although Barker’s hypothesis is well-established, this is the first study to explore its causal pathway using a rigorous, multi-time-point epigenetic MR framework with multi-omic integration. This represents a major advancement over previous observational and DNAm studies. The Barker-consistent hypothesis-free discovery approach identified novel and known candidate genes that, with future validation, may serve as therapeutic targets, identify high-risk individuals, and support clinical trial recruitment.

Barker’s hypothesis posits that adult cardiometabolic diseases result from adverse *in utero* exposures [1]. Natural famine studies linked intrauterine malnutrition to increased adult cardiometabolic syndrome risk [2]. Recent observational studies and meta-analyses reported an inverse relationship between birthweight and adult cardiometabolic disease risk [3–5], with growing evidence supporting a U-shaped relationship between birthweight and cardiovascular disease (CVD) risk [6,7]. Clinically, low birthweight is recognised as a surrogate biomarker of adverse intrauterine exposures and foetal programming for adult diseases [8,9], with potential for CVD risk stratification [10]. The hypothesis evolved into the Developmental Origins of Health and Disease framework, encompassing more intrauterine exposures and long-term health outcomes [9].

Although well-established, the mechanisms of Barker’s hypothesis remain poorly understood. One explanation, the thrifty-phenotype hypothesis, suggests dysregulation of cardiovascular, immune, and hypothalamic-pituitary-adrenal axis systems – adaptive for foetal survival during malnutrition – may be maladaptive in postnatal nutrient-rich environments [9]. Another is genetic pleiotropy, whereby variants influence beneficial foetal traits and later-life disease risk [9]. However, this theory alone is insufficient, as the rising prevalence of non-communicable diseases outpaces the rate of genetic variant-driven natural selection [3].

Adaptive traits may arise from developmental plasticity driven by epigenetic programming. DNA methylation (DNAm) is the most extensively studied epigenetic mechanism due to its relative stability [11]. While partially genetically determined, DNAm is highly responsive to perinatal environmental exposures, contributing to developmental plasticity and potentially serving as a biomarker or therapeutic target [12–15].

Initial epigenetic studies on birthweight effects employed a candidate-gene approach. Hypothalamic-pituitary-adrenal axis-related genes, including *NR3C1*, were differentially methylated in neonates exposed to maternal stress [16]. A more recent hypothesis-free epigenome-wide association study (EWAS) validated these findings and uncovered additional DNAm signals [17]. Adverse intrauterine exposures were linked to altered DNAm at genes involved in diverse processes, including metabolic, neuronal, and endocrine regulation [16]. A cross-sectional Dutch famine study retrospectively identified DNAm changes in genes enriched in growth and metabolism [2]. However, causal inference was constrained by unmeasured confounders and a lack of longitudinal data.

To establish epigenetic changes as potential causal mediators, three key criteria must be met: demonstrate interindividual epigenetic variability in early life resulting from adverse intrauterine exposures; confirm epigenetic changes precede disease onset; and determine the functional relevance of specific epigenetic modifications to the disease of interest [18]. Meeting these criteria is challenging with conventional observational studies, often requiring extensive time and resources for longitudinal data collection. Randomised controlled trials – the gold standard for causality – are not ethically feasible for studying adverse intrauterine exposures [19]. Thus, triangulation with alternative study designs is needed.

Epigenetic Mendelian randomisation (MR) addresses these limitations by leveraging genetic instruments to infer causality [20], satisfying three key criteria: Mendel’s laws enable randomisation of birthweight-associated epigenetic changes at birth, demonstrating interindividual variability in early life; examining causal links between pre-adulthood epigenetic changes and adverse adult CVD traits ensures temporal precedence; and assessing genes near persistently methylated loci with omics data supports their functional relevance and therapeutic potential for CVD. Previous MR studies only linked birthweight or maternal genetic influences on birthweight to cardiovascular outcomes [21,22]. Here, we aimed to extend this by applying epigenetic MR with multi-omics interrogation to investigate epigenetic mechanisms underpinning Barker’s hypothesis, identifying potential biological pathways for intervention.

We aimed to identify DNAm signatures as potential mediators between birthweight and adverse adult CVD traits using MR; to evaluate whether significant DNAm changes persist throughout key developmental stages: birth, childhood, and adolescence; and to establish the biological and functional significance of identified DNAm signatures by integrating omics data.

## METHODS

We employed methylation quantitative trait loci (mQTLs) [23] to proxy DNAm levels at birthweight-associated CpG sites (BW-CpGs), identified through EWAS. As low birthweight is a surrogate for adverse intrauterine exposures, BW-CpGs represent early-life signatures correlating with the intrauterine environment. In our primary analysis, we conducted epigenetic MR to assess the effects of DNAm at birth on adverse CVD traits, and carried out sensitivity analyses to assess association robustness. In secondary epigenetic MR, we examined DNAm measured in childhood and adolescence to identify persistent changes. Finally, we integrated omics data into an adapted scoring system to hypothesise biological mechanisms and prioritise candidate genes [24]. We followed the STROBE-MR reporting guidelines (Table S1 in the **Online Supplementary Document**).

The MR analysis infers causality when variants are: strongly associated with the exposure (relevance); independent of confounders of the exposure-outcome relationship (independence); and influence the outcome solely through the exposure (exclusion restriction) [25].

### Data sources

#### Exposures and genetic instruments

We selected two birthweight EWAS from the EWAS Catalog (Table S2 in the **Online Supplementary Document**) [17,26,27]. We chose BW-CpGs with Bonferroni-corrected significance thresholds (*P* < 1.06 × 10^−7^ [17]; *P* < 1.03 × 10^−3^ [26]). For BW-CpGs in both studies, we prioritised Küpers *et al.* [17] due to the larger sample size. Both EWASs analysed neonatal blood DNAm in European-ancestry individuals using the Illumina Infinium HumanMethylation450 BeadChip assay [28], and gene annotations used the corresponding Manifest file.

We obtained mQTLs for each independent BW-CpG from the mQTLdb [23] based on the Accessible Resource for Integrative Epigenomic Studies cohort (n = 1108 mother-offspring pairs). DNAm was measured in cord blood at birth (mean gestational age = 40 weeks) and peripheral blood during childhood (mean age = 7.49 years) and adolescence (mean age = 17.14 years), using the Illumina Infinium HumanMethylation 450 BeadChip array [23]. The discovery of mQTLs was adjusted for estimated white blood cell counts using a validated methylation-based algorithm across all time points, ensuring genetic effects on DNAm are independent of cellular composition and supporting persistence conclusions. For each BW-CpG at each time point, we used *cis*-mQTLs (within 1 Mb) [29] reaching genome-wide significance (*P* < 5 × 10^−8^) as proxies for DNAm levels. Independent mQTLs were identified using linkage disequilibrium (LD) clumping (r^2^<0.001, 10,000 kb window) with the 1000 Genomes European reference panel [30]. We excluded mQTLs outside 1 Mb of the BW-CpG and those removed by LD clumping.

#### Outcomes

We selected nine lipid traits and cardiovascular endpoints from a genome-wide association study (GWAS), based on established observational associations with low birthweight [3,7,31]. These included high-density lipoprotein cholesterol (HDL-C) [32], low-density lipoprotein cholesterol (LDL-C) [32], total cholesterol [32], and triglyceride levels [32]; coronary artery disease (CAD) [33], essential hypertension [34], any stroke (AS) [35], ischaemic stroke (IS) [35], and small-vessel stroke (SVS) [35] (Table S2 in the **Online Supplementary Document**). Sample sizes ranged from 98,048 (for SVS) to 547,261 (for CAD). Outcome datasets were from European-ancestry participants and excluded Accessible Resource for Integrative Epigenomic Studies cohort, minimising sample overlap.

#### Statistical analyses

Power calculations showed that given large GWAS sample sizes and assuming mQTLs can explain 15% of DNAm variance, our epigenetic MR analyses would have sufficient power to detect effect sizes for lipid traits and cardiovascular endpoints (Appendix S2 and Table S3 in the **Online Supplementary Document**).

Aligning with Barker’s hypothesis, low birthweight is associated with elevated LDL-C, total cholesterol, triglycerides, and cardiovascular risk (Figure S1 in the **Online Supplementary Document**). We confirmed consistency with this inverse association by ensuring effect estimates in step one (*α*) and step two (*β*) were in opposite directions for these outcomes. For HDL-C levels, we required steps one and two aligned, ensuring positive overall association. While BW-CpG selection from EWAS was hypothesis-free with respect to loci, lipid and cardiovascular outcomes and directional filtering were hypothesis-informed.

#### Primary analysis

We aligned *cis*-mQTL alleles using the harmonise_data function from the TwoSampleMR package to ensure consistent effect directions for BW-CpGs and outcomes. DNAm effects at each BW-CpG were assessed with Wald ratio or inverse variance weighted methods. We applied false discovery rate (FDR) correction for multiple testing (FDR-corrected *P* < 0.05). Instrument strength at each time point was assessed using F-statistics, with *F* *>*10 indicating strong instruments.

#### Sensitivity analysis

We conducted Steiger testing (for all associations), colocalisation (for associations with 1–2 mQTLs), and Cochran’s Q test along with pleiotropy-robust methods (for associations with ≥2 mQTLs). Steiger testing was applied to confirm the evaluated causal direction was appropriate for the selected mQTLs (*P* < 0.05). We used colocalisation (coloc.abf) to assess the probability that the exposure and outcome share a causal variant within 200 kb of the mapped gene or BW-CpG (posterior probability >0.8) [36]. Default prior probabilities were *p*_1_ = *p*_2_ = 1 × 10^−4^ and *p*_12_ = 1 × 10^−5^, where *p*_1_ is the probability the variant is causal for the BW-CpG, *p*_2_ for the outcome, and *p*_12_ for both. We employed Cochran’s Q test to evaluate significant heterogeneity among instruments (*P* < 0.05). Pleiotropy-robust methods (weighted median and MR-Egger) were used to assess potential horizontal pleiotropy, with directionally consistent effect sizes and inverse variance weighting indicating robust associations. Without full summary statistics for step one in two-step mediation MR, we used birthweight EWAS and did not perform multivariable MR (Appendix S1 in the **Online Supplementary Document**).

#### Persistent methylation and multi-omics interrogation

First, we repeated the primary and sensitivity analyses using mQTLs from childhood and adolescence to identify persistent DNAm changes. Second, we adapted a multi-omics approach for exploratory prioritisation to integrate evidence across omics layers [24]. We scored BW-CpGs from 0 to 5 based on: significant MR associations with ≥2 CVD traits; associations with Barker’s hypothesis traits in the EWAS Catalog [37] and EWAS Atlas [38]; presence of expression quantitative trait methylation (eQTM) data in the BIOS eQTM dataset [39] linking BW-CpGs to gene expression; the mQTL acting as an expression quantitative trait locus (eQTL) for the same gene in eQTLGen [40]; and associations of the mQTL with other traits in the GWAS Catalog [41] and the IEU GWAS database [42]. An arbitrary cutoff of four suggested consistent evidence across most omics layers, supporting functional relevance to Barker’s hypothesis and prioritising genes for future research (Appendix S1 in the **Online Supplementary Document**).

#### Software and Packages

We used *R*, version 4.3.1 (R Core Team, Vienna, Austria) for all analyses. Specifically, we used the ‘TwoSampleMR’ (version 0.6.8) package for MR analyses, Cochran’s Q test, pleiotropy-robust methods, and Steiger testing, ‘ieugwasr’ (version 1.0.1) package for LD clumping and retrieval of outcome summary statistics, and ‘coloc’ (version 5.2.3) package for Bayesian colocalisation.

## RESULTS

For the primary analysis, we included 261 BW-CpGs with 204 mQTLs measured at birth. All mQTLs had *F>*10 (median *F* at birth = 56.04, childhood = 84.03, adolescence = 80.53), indicating strong instruments. Epigenetic MR identified 230 associations between DNAm at BW-CpGs and adult cardiovascular traits consistent with Barker’s hypothesis. All 230 associations passed Steiger testing, and 42 remained robust across additional sensitivity analyses. Trajectory and sensitivity analyses of BW-CpGs from the primary analysis revealed three persistently methylated BW-CpGs mapped to *ASGR1* and *KDM2B* from birth through adolescence, with significant and robust associations. Multi-omics interrogation prioritised two BW-CpGs at *ASGR1* with an omics score of four (Figures S2 and S3 and Tables S4–9 in the **Online Supplementary Document**).

### Primary analysis

Epigenetic MR provided evidence of a causal effect of DNAm at BW-CpGs at birth on four adult lipid traits and five cardiovascular endpoints. In total, 230 of 1755 associations were significant (FDR-corrected *P* < 0.05) and were consistent with Barker’s hypothesis; this involved 106 of 261 studied BW-CpGs (78 mapped to genes and 28 unmapped) (Table S5 in the **Online Supplementary Document**). Most associations were for CAD (n = 42), hypertension (n = 37), and lipid traits, including HDL-C (n = 34), triglycerides (n = 30), LDL-C (n = 23), and total cholesterol (n = 21). Stroke subtypes – AS (n = 15), IS (n = 12), and SVS (n = 16) – had fewer associations, likely due to lower statistical power from smaller sample sizes and an imbalanced case-control ratio, limiting detection of modest epigenetic effects on binary outcomes (Table S3 in the **Online Supplementary Document**). The median odds ratio (OR) ranged from 0.942 for SVS to 1.027 for CAD (Appendix S3 in the **Online Supplementary Document**).

**Figure 1:**
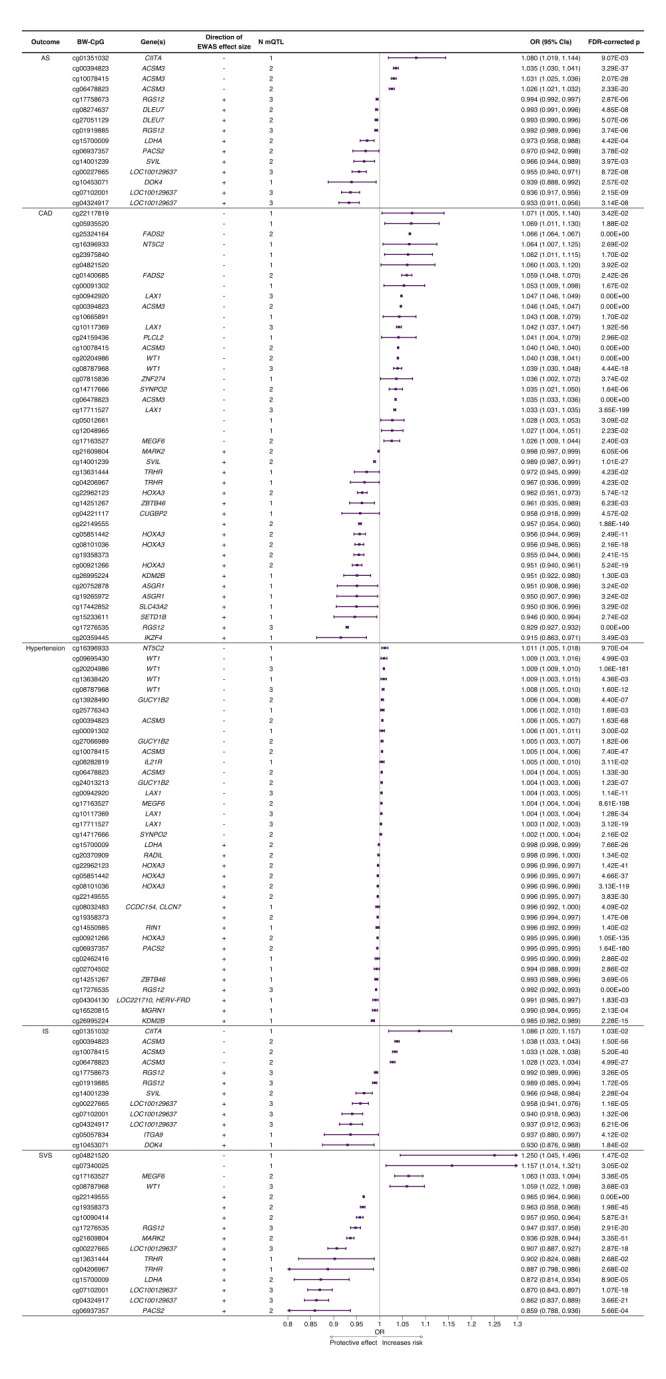
A forest plot of significant MR associations between BW-CpGs measured at birth and lipid traits consistent with Barker’s hypothesis. BW-CpG – birthweight-associated CpG site, CI – confidence interval, EWAS – epigenome-wide association study, FDR – false discovery rate, HDL-C – high-density lipoprotein cholesterol, LDL-C – low-density lipoprotein cholesterol, mQTL – methylation quantitative trait locus.

For lipid traits, we observed significant associations with 34 (14.7%) BW-CpGs for HDL-C, 23 (10%) for LDL-C, 21 (9.1%) for total cholesterol, and 30 (13%) for triglyceride levels, all aligning with Barker’s hypothesis (**Figure 1**; Table S5 in the **Online Supplementary Document**). The most pronounced increase in lipid levels was between DNAm at cg25324164 (*FADS2*) and total cholesterol (*β* = 0.105; 95% confidence interval (CI) = 0.070, 0.140; FDR-corrected *P* = 4.34 × 10^−9^). The greatest decrease in lipid levels was observed between DNAm at cg19265972 (*ASGR1*) and LDL-C (*β* = −0.071; 95% CI = −0.084, −0.057; FDR-corrected *P* = 4.87 × 10^−23^).

**Figure 2:**
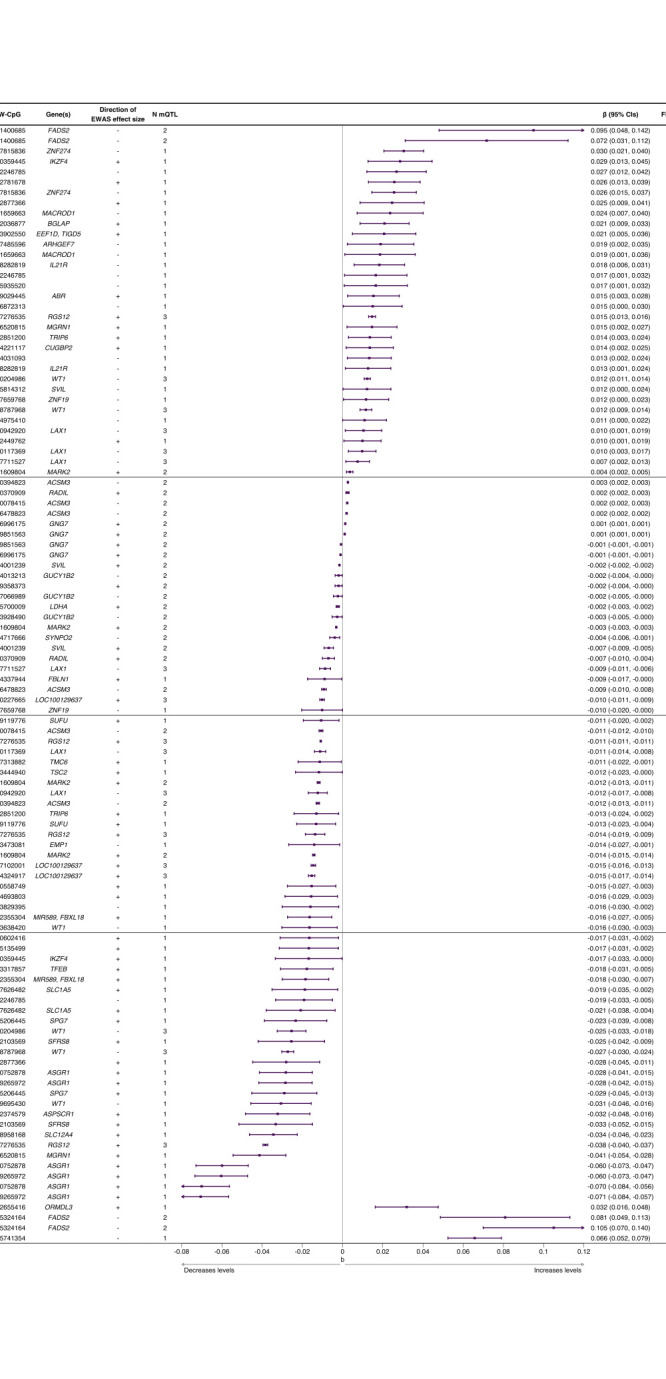
A forest plot of significant MR associations between BW-CpGs measured at birth and cardiovascular outcomes consistent with Barker’s hypothesis. AS – any stroke, BW-CpG – birthweight-associated CpG site, CAD – coronary artery disease, CI – confidence interval, EWAS – epigenome-wide association study, FDR – false discovery rate, IS – ischaemic stroke, mQTL – methylation quantitative trait locus, OR – odds ratio, SVS – small-vessel stroke.

For cardiovascular endpoints, we identified significant associations between DNAm at 15 (6.5%) BW-CpGs and AS, 42 (18.3%) BW-CpGs and CAD, 37 (16%) BW-CpGs and hypertension, 12 (5.2%) BW-CpGs and IS, and 16 (7%) BW-CpGs and SVS, all consistent with Barker’s hypothesis (**Figure 2**; Table S5 in the **Online Supplementary Document**). The greatest increase in cardiovascular risk was observed for DNAm at cg04821520 (unmapped) and SVS (OR = 1.250; 95% CI = 1.045, 1.496; FDR-corrected *P* = 1.47 × 10^−2^). The most notable decrease in cardiovascular risk was found between DNAm at cg06937357 (*PACS2*) and SVS (OR = 0.859; 95% CI = 0.788, 0.936; FDR-corrected *P* = 5.66 × 10^−4^).

### Sensitivity analyses

All 230 significant associations passed Steiger testing (*P* < 0.05), suggesting correct causal direction. For 107 single-instrument associations, 11 (5.7%) passed colocalisation (posterior probability >0.8), indicating shared causal variants likely drive these effects – HDL-C (n = 1), LDL-C (n = 3), total cholesterol (n = 3), triglycerides (n = 3), and hypertension (n = 1) (Table S6 in the **Online Supplementary Document**). It is important to note that we performed 112 colocalisation tests as five BW-CpG associations mapped to two genes. For 80 associations with two *cis*-mQTLs, none passed colocalisation (posterior probability >0.8). All 43 associations with ≥2 mQTLs showed no evidence of heterogeneity (*i.e.* insignificant Cochran’s Q), and 31 (72.1%) exhibited consistent effect directions across inverse variance weighted and pleiotropy-robust methods, suggesting minimal bias from horizontal pleiotropy. These included HDL-C (n = 4), LDL-C (n = 3), total cholesterol (n = 1), triglycerides (n = 5), CAD (n = 4), hypertension (n = 4), AS (n = 3), IS (n = 5), and SVS (n = 2) (Table S5 in the **Online Supplementary Document**). Of the 42 associations passing all sensitivity analyses, 18 BW-CpGs mapped to 8 genes: *ASGR1*, *KDM2B*, *LAX1*, *LOC100129637*, *RGS12*, *SLC12A4*, *WT1*, and *ZNF274*.

### Trajectory analysis and multi-omics interrogation

We identified BW-CpGs with consistent methylation patterns and robust associations from birth through adolescence, mapped to *ASGR1* (cg19265972, cg20752878) and *KDM2B* (cg26995224) (Tables S7 and S8 and Figure S4 in the **Online Supplementary Document**).

We retrieved additional data for the three BW-CpGs and their corresponding mQTLs from omics data to generate an omics score for each BW-CpG (Table S9 in the **Online Supplementary Document**). The two BW-CpGs mapped to *ASGR1* reached the predefined omics score threshold of four. No eQTMs were available for the three BW-CpGs. However, the corresponding eQTL for mQTL at cg19265972 and cg20752878 (both mapping to *ASGR1*) showed reduced gene expression, suggesting the mQTL is linked to decreased *ASGR1* expression.

## DISCUSSION

**Figure 3:**
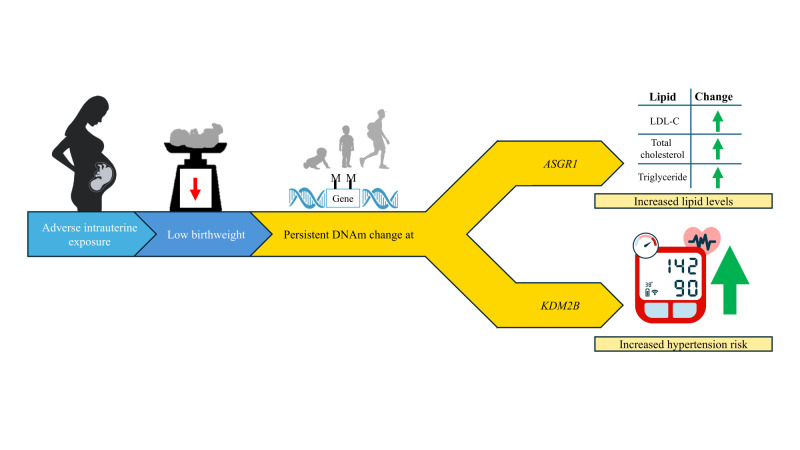
Overview of key findings. DNAm – DNA methylation, LDL-C – low-density-lipoprotein cholesterol.

We employed an MR approach for hypothesis-free discovery of CpG candidates within a Barker-consistent framework to provide insights into the mechanistic role of DNAm in Barker’s hypothesis (**Figure 3**). We identified significant and robust associations between BW-CpGs and CVD traits through primary and sensitivity analyses. Secondary analyses, including trajectory analysis and multi-omics interrogation, highlighted *ASGR1* as a key candidate gene for intervention, setting this study apart from previous Developmental Origins of Health and Disease and epigenetic MR literature and supporting epigenetics as a potential mediator in Barker’s hypothesis with clinical implications.

Through MR using birth mQTLs, we found significant and robust associations between BW-CpGs and all nine outcomes following stringent sensitivity analyses. However, trajectory analysis showed no persistent associations with CAD or stroke across development – possibly due to limited power for binary traits – whereas associations with hypertension, LDL-C, total cholesterol, and triglycerides align with prior studies [4,5,43,44]. Our findings implicated DNAm at *ASGR1* and *KDM2B* in this birthweight-CVD pathway. Notably, *ASGR1* had an omics score of four. It encodes a hepatically expressed asialoglycoprotein receptor subunit, regulating serum glycoprotein homeostasis, and is being explored as a therapeutic target for CVD.

Increased DNAm at two *ASGR1* BW-CpGs was consistently associated with lower LDL-C (cg19265972, cg20752878), total cholesterol (cg19265972, cg20752878), and triglyceride levels (cg20752878). Although no eQTM data are currently available, the *cis*-mQTL also functions as an eQTL for *ASGR1* (Z = −9.570), suggesting shared genetic regulation of methylation and expression at this locus. Additionally, the mQTL used to proxy DNAm at these sites was linked with lipid traits in GWAS datasets, further reinforcing *ASGR1*’s role in lipid metabolism.

In the context of Barker’s hypothesis, *ASGR1* is a potential therapeutic target for adverse CVD traits. Deficiency of *ASGR1* inhibits mTORC1 signalling, reduces cholesterol synthesis, and up-regulates proteins promoting cholesterol transport and excretion. A recent study using pig models demonstrated that *ASGR1* deficiency down-regulates 3-hydroxy-3-methylglutaryl-CoA reductase and LDL-C receptors [45]. A large population-based study in Iceland further supported this finding, showing *ASGR1* loss-of-function variants were associated with lower non-HDL cholesterol and reduced CAD risk [46,47]. Promising results are also emerging from ongoing drug development efforts targeting *ASGR1*. The causal effects of *ASGR1* inhibition were initially confirmed by an MR study investigating loss-of-function variants [48]. Compared to established lipid-lowering therapies, *ASGR1* inhibition produced similar reductions in total cholesterol and CAD risk – and even stronger reductions in triglyceride levels. To replicate these genetic effects, a monoclonal antibody (AMG 529) blocking *ASGR1* is currently in development. A recent phase I clinical trial reported no significant dose-related adverse effects, supporting its continued investigation [49].

We identified *ASGR1* as a biologically plausible and potentially targetable mediator in the birthweight-CVD pathway. Validation in independent longitudinal cohorts of diverse ancestries – with measured DNAm, lipid, and cardiovascular outcomes – is needed before clinical translation. This would support the consideration of persistent epigenetic signatures at *ASGR1* and *KDM2B* for screening, clinical trial recruitment, and therapeutic targets for high-risk individuals for developing CVD.

### Strengths and limitations

We made efforts to mitigate the limitations of observational studies and to provide causal evidence for epigenetics as a potential mediator in Barker’s hypothesis [18]. We used large GWASs, validated causal directions, applied stringent sensitivity analyses to minimise MR assumptions violations, and interrogated omics datasets to explore potential biological mechanisms.

However, several limitations remain. Due to dataset restrictions, EWASs served as proxies for the first step of the two-step mediation MR analysis. Full access to summary statistics would enable future epigenetic studies to conduct formal mediation MR. Nonetheless, effect sizes from EWASs and MR were directionally consistent with Barker’s hypothesis. The MR analysis uses genetic instruments, which reflect average lifetime effects. However, MR effect sizes may not directly translate to clinical outcomes, requiring further research to bridge this gap. Colocalisation was restricted to associations with fewer than three mQTLs. The non-colocalised associations, which may have directionally consistent effect sizes across pleiotropy-robust methods, should be interpreted as supporting rather than evidence of potential mediators, as LD confounding cannot be excluded. Nevertheless, 18 BW-CpGs passed the stringent sensitivity analyses, representing the highest-confidence findings. No universally accepted methodology exists for gene prioritisation in Barker’s hypothesis. Our adapted scoring system is exploratory, and future functional assays are needed to validate candidate genes. The limited availability of DNAm data also constrains the generalisability of our findings. While GWAS exist for related traits such as head circumference and placental weight, the absence of corresponding EWAS prevented DNAm mediation analysis for these traits. Although many CVD traits affect multiple tissues, including the heart and liver, our analysis was restricted to blood-based DNAm data. The *ASGR1* is expressed in the liver, where it maintains glycoprotein homeostasis. While blood DNAm offers non-invasive data, it may partially capture hepatic epigenetic regulation. However, one criterion for confirming DNAm as potential causal mediators is to establish the temporal sequence of epigenetic changes, thus confirming they precede disease onset [18]. Currently, blood is the only tissue with DNAm profiled across three developmental time points. Extending this work to tissue-specific methylation data would strengthen tissue-relevant inference for *ASGR1*. Regarding population diversity, all analyses were based on European-ancestry participants, limiting the applicability of our results to other populations. Moreover, the univariable MR approach assumes a linear exposure-outcome relationship, while observational studies suggested a U-shaped association between birthweight and adult CVD traits [6,7]. While nonlinear MR methods exist [50], they require individual-level statistics [19], which are unavailable for this study. We derived mQTLs measured at birth from offspring cord blood, primarily reflecting foetal genetic influences on DNAm. However, maternal genetic variants may influence adverse intrauterine exposures and birthweight-related DNAm patterns [51], yet limited data on maternal genetic contributions to offspring DNAm make it difficult to disentangle foetal from maternal influences. Future longitudinal epigenetic studies should incorporate maternal genetic effects.

## CONCLUSIONS

Although prior MR studies examined birthweight or maternal genetic contributions to cardiovascular traits, none employed an epigenetic MR framework that simultaneously investigated BW-CpGs across three developmental time points and integrated multi-omics scoring to prioritise candidate genes. This study represents the first systematic effort to identify DNAm at BW-CpGs as potential mediators in Barker’s hypothesis, addressing limitations of prior DNAm studies. Using a hypothesis-free discovery of CpG candidates within a Barker-consistent framework, we identified *ASGR1* as the primary candidate mediating adverse CVD traits in line with Barker’s hypothesis. Low birthweight individuals – at elevated CVD risk – could be screened for DNAm signatures at *ASGR1* and *KDM2B*, identifying them as potential candidates for future clinical trials.

## Additional material


Online Supplementary Document

## Data Availability

**Data availability:** The datasets supporting the conclusions of this article are publicly available EWAS and GWAS summary statistics. Code used for analysis can be made available upon reasonable request.
