# Supplementary material for: DNA methylation informs adult cardiovascular risk and prevention for low birthweight individuals
Source: J Glob Health. 2026 Aug 14;16:04242. doi: 10.7189/jogh.16.04242 (PMC13474067; doi:10.7189/jogh.16.04242)
Supplement: Supplementary file 1 [file jogh-16-04242-s001.zip › jogh-16-04242-s001.pdf]

Supplement to: Tang J, Ng N, Kwok A, Mak C, Cheung YF, Chung B. DNA methylation informsadult cardiovascular risk and prevention for low birthweight individuals. J Glob Health. 2026;16:04242.

SUPPLEMENTARY APPENDIX S1.

Study design

This study explored epigenetics as a mechanism to explain the impact of low birthweight on adverse cardiovascular traits in adulthood, as described in Barker's hypothesis [1]. Causality was inferred with epigenetic Mendelian randomisation (MR) analysis by employing methylation quantitative trait loci (mQTLs) as genetic variants proxying birthweight-associated CpG sites (BW-CpGs). Summary statistics were obtained from mQTLdb. The same mQTLs were used as genetic variants to proxy cardiovascular traits as outcomes, with no sample overlap across mQTL and cardiovascular trait datasets.

We selected nine lipid traits and cardiovascular endpoints from GWAS, based on their established observational associations with low birthweight [2–4]. These included high-density lipoprotein cholesterol (HDL-C), low-density lipoprotein cholesterol (LDL-C), total cholesterol, and triglyceride levels; coronary artery disease (CAD), essential hypertension, any stroke (AS), ischaemic stroke (IS), and small-vessel stroke (SVS). We selected these datasets as they excluded individuals from the exposure dataset, minimising sample overlap bias. For lipid traits, effect sizes estimated using MR represent a change in the trait per unit increase in DNAm; for cardiovascular outcomes, they indicate a change in disease odds per unit increase in DNAm.

Two-step mediation MR involves carrying out MR in two steps to investigate the relationship between an exposure on an outcome through a mediator. For a trait to be considered as an outcome in MR, the full set of associations is needed. However, since the reported maximum p-value from mQTLdb was 1×10^{-4}, comprehensive two-step mediation MR was not technically possible. Instead, EWAS were used to assess associations between differentially methylated BW-CpGs and birthweight as a proxy for step one [3–5]. MR was then carried out in step two with BW-CpGs as exposures to determine the causal effects of DNAm on cardiovascular traits. Depending on the number of available mQTLs, we employed either Wald ratio or inverse variance weighted (IVW) methods.

Mendelian randomisation assumptions

Causal inference via MR holds if the genetic variants satisfy three assumptions: 1) the variants are strongly associated with the exposure of interest (relevance); 2) the variants are not associated with confounders of the exposure to outcome association (independence); and 3) the variants only influence the outcome through the exposure of interest, with no direct effect on the outcome (exclusion restriction) [6].

The relevance assumption can be evaluated using F-statistics. Evaluation of independence and restriction assumptions may be violated due to horizontal pleiotropy, linkage disequilibrium (LD), and population substructure [7]. Cochran's Q can test for potential pleiotropy. Pleiotropy-robust MR estimators can evaluate causal relationships under relaxed assumptions [8,9].

Assessing violations of MR assumptions

F-statistics

To assess instrument strength, F-statistics for each mQTL at each timepoint were calculated using Eq. 1 [10].

$$F = \frac{\beta^2}{SE^2} \tag{1}$$

This is where *ß* is the effect size of the mQTL, and *SE* is the standard error. F-statistics greater than 10 indicate a strong association between mQTLs and BW-CpGs (relevance).

Steiger testing

After carrying out epigenetic MR for each possible CpG-outcome pair, we performed the Steiger test with the selected genetic variants to confirm the direction of analysis, as a support for the MR assumptions. This method does not require a full understanding of the underlying biology of each genetic variant that is used. Correlations between genetic variants and BW-CpGs, and between genetic variants and outcomes are estimated. If the correlation between genetic variants and BW-CpGs exceeds the correlation between genetic variants and outcomes and $p < 0.05$, the direction of causality being investigated is confirmed [11].

Colocalisation

Most MR associations only had one or two mQTLs available, so Cochran's Q test and pleiotropy-robust methods could not be conducted for these associations. As an alternative, colocalisation was performed on statistically significant BW-CpG—outcome associations (defined as false discovery rate (FDR) corrected $p < 0.05$). Colocalisation aims to identify whether the association investigated is due to LD, this is a potential reason for horizontal pleiotropy which violates MR assumptions (independence, exclusion restriction). Colocalisation involves performing hypothesis testing to assess the relationship between BW-CpGs and outcomes at loci in a genetic region. This was defined as ±200 kb from the BW-CpG or the nearest gene, if available. Gene mapping was done using the Infinium HumanMethylation450 Manifest file [12].

Colocalisation involves testing these hypotheses: H_0, there is no genetic association in the region for either BW-CpG or the outcome; H_1, only the BW-CpG has a genetic association in the region; H_2, only the outcome has a genetic association in the region; H_3, both the BW-CpG and outcome are associated, but with different causal variants; and H_4, both the BW-CpG and outcome are associated and share a causal variant.

Accepting H_4 for a BW-CpG—outcome association indicates that the exclusion restriction assumption is not violated by LD. H_4 does not distinguish between two possibilities: either the variant is causal for both the BW-CpG and the outcome, or the

variant is causal for the BW-CpG which in turn affects the outcome. Combining colocalisation evidence with MR associations thus ensured that the causal variant acts through BW-CpG to influence the outcome.

The Approximate Bayes Factor was used to calculate posterior probabilities for each genetic variant, which serves as a proxy for the association between the BW-CpG and the outcome [13]. Default prior probabilities were used: $p_1 = p_2 = 1\times10^{-4}$ and $p_{12} = 1\times10^{-5}$, where p_1 is the probability that the genetic variant is causal for the BW-CpG, p_2 is the probability that the genetic variant is causal for the outcome, and p_{12} is the probability that the genetic variant is causal for both the BW-CpG and the outcome. These prior probabilities were used to compute the posterior probabilities for each hypothesis. Posterior probability cutoff is defined as PP.H4 > 0.8 [13]. Associations that exceed this threshold are said to pass colocalisation. Additional information may be found in other literature [13,14].

Cochran's Q test

For the remaining MR associations that had more than two mQTLs, Cochran's Q test was used to evaluate potential heterogeneity across the effects of each genetic instrument due to pleiotropy. Q-statistics that are significantly larger than the degrees of freedom ($p < 0.05$) suggest possible violation of MR assumptions (independence, exclusion restriction).

Pleiotropy-robust methods

In addition, weighted median and MR-Egger were used to evaluate the extent of horizontal pleiotropy by relaxing MR assumptions (independence, exclusion restriction). The weighted median method allows for a maximum of 50% of mQTLs to be invalid whilst ensuring an effect size estimate that is directionally consistent with the estimate from the inverse variance weighted (IVW) method [8]. The MR-Egger method yields an estimate that is directionally consistent with the IVW estimate under the condition that either no horizontal pleiotropy exists, or it follows the "Instrument Strength Independent of Direct Effect" (InSIDE) assumption [9]. This assumption is satisfied when the mQTL—BW-CpG associations are independent from the pleiotropic effects of mQTLs. Robust associations were indicated by directionally consistent effect sizes across IVW and these pleiotropy-robust methods.

Multi-omics interrogation

A multi-omics approach was used to investigate mechanistic links between genetics, epigenetics, transcriptomics, and phenotypes in the context of Barker's hypothesis. This is similar to an approach taken in a recent study [15]. The datasets used were from epigenome-wide association studies (EWAS) and genome-wide association studies (GWAS), and are expression quantitative methylation (eQTM) and expression quantitative loci (eQTL) datasets. Significant BW-CpGs that passed sensitivity analyses and their mQTLs from epigenetic MR were used in multi-omics interrogation.

Adapting the methodology by Wang et al (2023) [15], we derived an omics score out of five for each BW-CpG. Satisfying each of the following criteria contributed one point to the omics score: (1) significant MR association with two or more CVD traits; (2) the BW-CpG was associated with traits related to Barker's hypothesis in EWAS, using the EWAS Catalog [16] and EWAS Atlas [17]; (3) an eQTM was available to link altered DNAm at the BW-CpG to expression of the mapped gene, using the BIOS eQTM dataset [18]; (4) the mQTL was an eQTL for the same gene, using the eQTLGen database [19]; and (5) the mQTL was reported in previous GWAS, using the GWAS Catalog [20] and the IEUGWAS database [21]. An arbitrary score of four suggested consistent evidence across literature across different omics traits. Additional information on the druggability of mapped genes was obtained from DrugBank [22].

EWAS datasets

The associations of BW-CpGs with other phenotypes from the EWAS Catalog [16] and EWAS Atlas [17] were examined. Data from these sources was accessed on 19 April 2024. Duplicated phenotypes were excluded, and the remaining phenotypes were recorded.

GWAS datasets

Associations of mQTLs with GWAS phenotypes were examined. Data used from the GWAS Catalog [20] and IEUGWAS database [21] were accessed on 19 April 2024. Associations with a significance threshold of $p < 5\times10^{-8}$ were selected, duplicate phenotypes were removed, and the remaining phenotypes were grouped. We recorded the remaining phenotypes and their groupings.

eQTM dataset

The BIOS eQTM dataset [18] contains peripheral blood measurements of eQTMs from four cohorts. We downloaded the dataset and searched for eQTMs associated with BW-CpG. We identified eQTMs that were in cis to the BW-CpG (within 10 kb of the transcription start site and the transcription end site) with FDR < 0.05 that mapped to the same gene. In addition, the effect size, the standard error, and FDR from the largest cohort were recorded of each eQTM.

eQTL dataset

eQTLGen [19] has data on measured eQTLs in peripheral blood. We searched for mQTLs in eQTLGen. We identified eQTLs that were within 1 Mb from the center of the gene (cis) with $p < 1\times10^{-5}$, and that mapped to the same gene. We also recorded the Z-score, effect allele, other allele, and FDR for each eQTL.

SUPPLEMENTARY APPENDIX S2.

A priori statistical power for the MR analyses was calculated using an online tool (https://sb452.shinyapps.io/power/) (Table S3 in the **Online Supplementary Document**). Each MR association was assessed using a Type I error rate of 0.05, the proportion of DNAm variance explained by mQTLs ($R^2 = 0.15$) [2], the sample size for each outcome, the case-control ratio for binary outcomes, and a range of assumed effect sizes for BW-CpGs on outcomes odds ratios ([ORs] from 0.84 [β = –0.17] to 1.16 [β = 0.15]). For example, in the case of any stroke (AS), statistical power exceeds 80% if the expected OR is ≤0.96 or ≥1.04. Power estimates for all MR associations are presented in Table S3 in the **Online Supplementary Document**.

SUPPLEMENTARY APPENDIX S3.

For lipid traits as outcomes, we found significant associations between 34 BW-CpGs and HDL-C, 23 BW-CpGs and LDL-C, 21 BW-CpGs and total cholesterol, and 30 BW-CpG and triglyceride levels that aligned with Barker's hypothesis (Table S4 in the **Online Supplementary Document**).

Of the 34 BW-CpG associations with HDL-C, DNAm at cg12655416 (*ORMDL3*) was associated with the largest increase in HDL-C (β: 0.032; 95% CIs: 0.016, 0.048; FDR-corrected $p < 0.001$). DNAm at cg09695430 (*WT1*) was associated with the largest decrease in HDL-C (β: –0.031; 95% CIs: –0.046, –0.016; FDR-corrected $p < 0.001$).

Of the 23 BW-CpGs associations with LDL-C, DNAm at cg25324164 (*FADS2*) was associated with the largest increase in LDL-C (β: 0.081; 95% CIs: 0.049, 0.113; FDR-corrected $p < 0.001$). DNAm at cg19265972 (*ASGR1*) was associated with the largest decrease in LDL-C (β: –0.071; 95% CIs: –0.084, –0.057; FDR-corrected $p < 0.001$).

Of the 21 BW-CpGs associations with total cholesterol, DNAm at cg25324164 (*FADS2*) was associated with the largest increase in total cholesterol (β: 0.105; 95% CIs: 0.070, 0.140; FDR-corrected $p < 0.001$). DNAm of cg19265972 (*ASGR1*) was associated with the largest decrease in total cholesterol (β: –0.060; 95% CIs: –0.073, –0.047; FDR-corrected $p < 0.001$).

Of the 30 BW-CpGs associated with triglycerides, DNAm at cg15741354 (not mapped to a gene) was associated with the largest increase of triglyceride levels (β: 0.066; 95% CIs: 0.052, 0.079; FDR-corrected $p < 0.001$). DNAm of cg16520815 (*MGRN1*) was associated with the largest decrease in triglycerides (β: -0.041; 95% CIs: -0.054, -0.028; FDR-corrected $p < 0.001$).

For cardiovascular outcomes, we found significant associations between 15 BW-CpGs and AS, 42 BW-CpGs and CAD, 37 BW-CpGs and hypertension, 12 BW-CpGs and IS, and 16 BW-CpGs and SVS that aligned with Barker's hypothesis (Table S4 in the **Online Supplementary Document**).

Of the 15 BW-CpGs associated with AS, cg01351032 (*CIITA*) was associated with the greatest increase in AS risk (OR: 1.080; 95% CIs: 1.019, 1.144; FDR-corrected p: 9.07×10^{-3}). DNAm at cg04324917 (*LOC100129637*) was associated with the greatest decrease in AS risk (OR: 0.933; 95% CIs: 0.911, 0.956; FDR-corrected $p < 0.001$).

Of the 42 BW-CpGs associated with CAD, DNAm at cg22117819 (not mapped to any gene) was associated with the largest increase in CAD risk (OR: 1.071; 95% CIs: 1.005, 1.140, FDR-corrected p: 3.42×10^{-2}). DNAm at cg20359445 (*IKZF4*) was associated with the largest decrease in CAD risk (OR: 0.915; 95% CIs: 0.863, 0.971, FDR-corrected p: 3.49×10^{-3}).

Of the 37 BW-CpGs associated with hypertension, cg16396933 (*NT5C2*) was associated with the greatest increase in hypertension risk (OR: 1.011; 95% CIs: 1.005,

1.018; FDR-corrected *p*: 9.70×10^{-4}). DNAm at cg26995224 (*KDM2B*) was associated with the largest decrease in hypertension risk (OR: 0.985; 95% CIs: 0.982, 0.989; FDR-corrected $p < 0.001$).

Of the 12 BW-CpGs associated with IS, cg01351032 (*CIITA*) was associated with the greatest increase in IS risk (OR: 1.086; 95% CIs: 1.020, 1.157; FDR-corrected *p*: 1.03×10^{-2}). DNAm at cg10453071 (*DOK4*) was associated with the largest decrease in IS risk (OR: 0.930; 95% CIs: 0.876, 0.988; FDR-corrected *p*: 1.84×10^{-2}).

Of the 16 BW-CpGs associated with SVS, cg04821520 (not mapped to any gene) was associated with the greatest increase in SVS risk (OR: 1.250; 95% CIs: 1.045, 1.496; FDR-corrected *p*: 1.47×10^{-2}). DNAm at cg06937357 (*PACS2*) was associated with the largest decrease in SVS risk (OR: 0.859; 95% CIs: 0.788, 0.936; FDR-corrected *p*: 5.66×10^{-4}).

SUPPLEMENTARY REFERENCES

1. Barker DJ, Osmond C. Infant mortality, childhood nutrition, and ischaemic heart disease in England and Wales. Lancet. 1986;327:1077–81. doi:10.1016/s0140-6736(86)91340-1

2. Min JL, Hemani G, Hannon E, Dekkers KF, Castillo-Fernandez J, Luijk R, et al. Genomic and phenotypic insights from an atlas of genetic effects on DNA methylation. Nat Genet. 2021;53:1311–1321. doi:10.1038/s41588-021-00923-x

3. Richardson TG, Zheng J, Davey Smith G, Timpson NJ, Gaunt TR, Relton CL, et al. Mendelian Randomization Analysis Identifies CpG Sites as Putative Mediators for Genetic Influences on Cardiovascular Disease Risk. Am J Hum Genet. 2017;101:590–602. doi:10.1016/j.ajhg.2017.09.003

4. Relton CL, Davey Smith G. Two-step epigenetic Mendelian randomization: a strategy for establishing the causal role of epigenetic processes in pathways to disease. Int J Epidemiol. 2012;41:161–176. doi:10.1093/ije/dyr233

5. Xu S, Li X, Zhang S, Qi C, Zhang Z, Ma R, et al. Oxidative stress gene expression, DNA methylation, and gut microbiota interaction trigger Crohn's disease: a multi-omics Mendelian randomization study. BMC Med. 2023;21:179. doi:10.1186/s12916-023-02878-8

6. Davies NM, Holmes MV, Davey Smith G. Reading Mendelian randomisation studies: a guide, glossary, and checklist for clinicians. BMJ. 2018;362:k601. doi: 10.1136/bmj.k601

7. Teumer A. Common Methods for Performing Mendelian Randomization. Front Cardiovasc Med. 2018;5:51. doi:10.3389/fcvm.2018.00051

8. Bowden J, Davey Smith G, Haycock PC, et al. Consistent Estimation in Mendelian Randomization with Some Invalid Instruments Using a Weighted Median Estimator. Genet Epidemiol. 2016;40:304–314. doi:10.1002/gepi.21965

9. Bowden J, Davey Smith G, Burgess S. Mendelian randomization with invalid instruments: effect estimation and bias detection through Egger regression. Int J Epidemiol. 2015;44:512–525. doi:10.1093/ije/dyv080

10. Bowden J, Del Greco MF, Minelli C, Davey Smith G, Sheehan NA, Thompson JR. Assessing the suitability of summary data for two-sample Mendelian randomization analyses using MR-Egger regression: the role of the I2 statistic. Int J Epidemiol. 2016;45:1961–1974. doi:10.1093/ije/dyw220

11. Hemani G, Tilling K, Davey Smith G. Orienting the causal relationship between imprecisely measured traits using GWAS summary data. PLoS Genet. 2017;13:e1007081. doi:10.1371/journal.pgen.1007081

12. Illumina. HumanMethylation 450 v1.2 Manifest File (CSV Format) [dataset]. 2013. Available:

https://support.illumina.com/downloads/infinium_humanmethylation450_product_files.html Accessed: 20 May 2025.

13. Zuber V, Grinberg NF, Gill D, Manipur I, Slob EAW, Patel A, et al. Combining evidence from Mendelian randomization and colocalization: Review and comparison of approaches. Am J Hum Genet. 2022;109:767–782. doi: 10.1016/j.ajhg.2022.04.001

14. Giambartolomei C, Vukcevic D, Schadt EE, Franke L, Hingorani AD, Wallace C, et al. Bayesian test for colocalisation between pairs of genetic association studies using summary statistics. PLoS Genet. 2014;10:e1004383. doi:10.1371/journal.pgen.1004383

15. Wang X, Campbell MR, Cho H-Y, Pittman GS, Martos SN, Bell DA. Epigenomic profiling of isolated blood cell types reveals highly specific B cell smoking signatures and links to disease risk. Clin Epigenetics. 2023;15:90. doi:10.1186/s13148-023-01507-8

16. Battram T, Yousefi P, Crawford G, Prince C, Babaei MS, Sharp G, et al. The EWAS Catalog: a database of epigenome-wide association studies. Wellcome Open Res. 2022;7:41. doi:10.12688/wellcomeopenres.17598.2

17. Xiong Z, Yang F, Li M, Ma Y, Zhao W, Wang G, et al. EWAS Open Platform: integrated data, knowledge and toolkit for epigenome-wide association study. Nucleic Acids Res. 2022;50:D1004–D1009. doi:10.1093/nar/gkab972

18. Bonder MJ, Luijk R, Zhernakova DV, Moed M, Deelen P, Vermaat M, et al. Disease variants alter transcription factor levels and methylation of their binding sites. Nat Genet. 2017;49:131–138. doi:10.1038/ng.3721

19. Vosa U, Claringbould A, Westra H-J, Bonder MJ, Deelen P, Zeng B, et al. Large-scale cis- and trans-eQTL analyses identify thousands of genetic loci and polygenic scores that regulate blood gene expression. Nat Genet. 2021;53:1300–1310. doi:10.1038/s41588-021-00913-z

20. Buniello A, MacArthur JAL, Cerezo M, Harris LW, Hayhurst J, Malangone C, et al. The NHGRI-EBI GWAS Catalog of published genome-wide association studies, targeted arrays and summary statistics 2019. Nucleic Acids Res. 2019;47:D1005–D1012. doi: 10.1093/nar/gky1120

21. Elsworth B, Lyon M, Alexander T, Liu Y, Matthews P, Hallett J, et al. The MRC IEU OpenGWAS data infrastructure. bioRxiv: 244293 [preprint]. 2020. Available: https://doi.org/10.1101/2020.08.10.244293 Accessed: 30 June 2025

22. Knox C, Wilson M, Klinger CM, Franklin M, Oler E, Wilson A, et al. DrugBank 6.0: the DrugBank Knowledgebase for 2024. Nucleic Acids Res. 2024;52:D1265–D1275. doi:10.1093/nar/gkad976

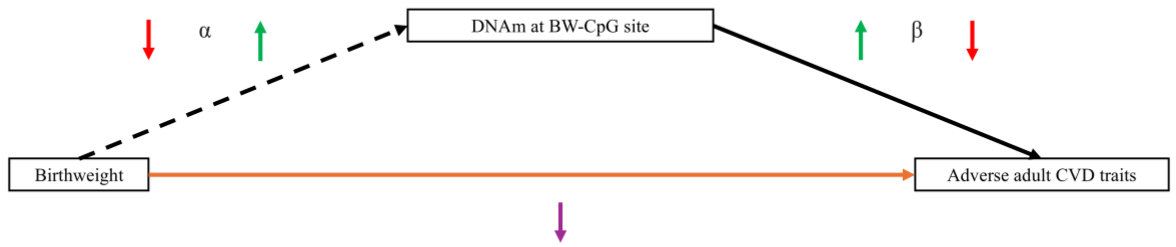

Figure S1. Epigenetic MR study design. This framework identified DNAm at BW-CpGs from EWASs and evaluated their causal relationship with adverse adult CVD traits. Orange arrow: established associations from previous studies. The direction of the product of α and β aligned with the hypothesised effect: a negative overall product (purple arrow) supports Barker's hypothesis (i.e. $+\alpha\times-\beta$ or $-\alpha\times+\beta$). α, association between birthweight and DNAm at individual BW-CpGs from EWAS; β, causal estimate from epigenetic MR analysis in this study; BW-CpG, birthweight-associated CpG site; CVD, cardiovascular disease; DNAm, DNA methylation; EWAS, epigenome-wide association studies.

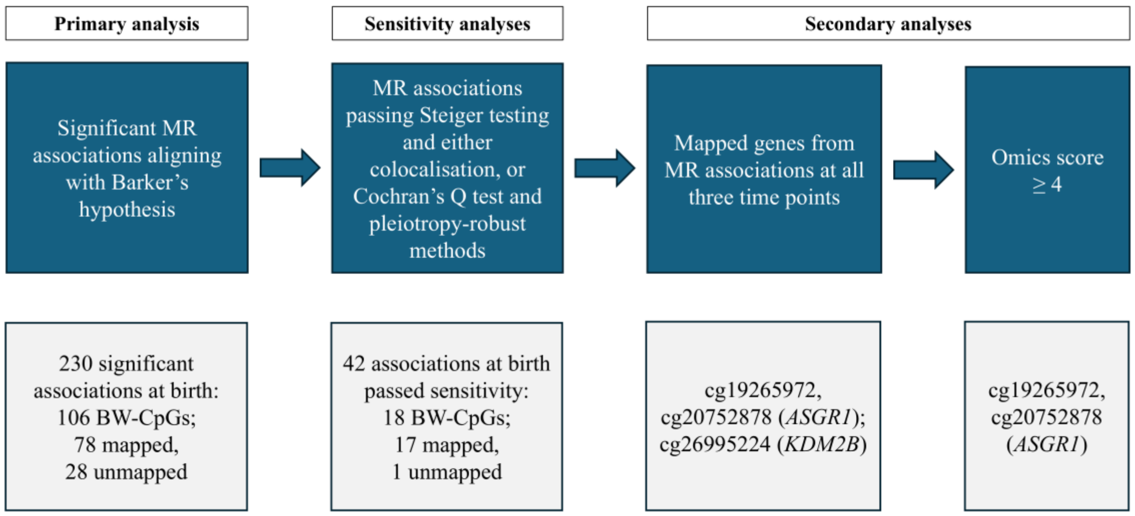

Figure S2. Overview of analytical stages and key findings. Supporting Barker's hypothesis, 230 significant associations between BW-CpGs and adverse adult CVD traits at birth were identified; 42 of these demonstrated robustness in sensitivity analyses. Secondary analyses prioritised one gene for further investigation. BW-CpGs, birthweight-associated CpG sites; MR, Mendelian randomisation.

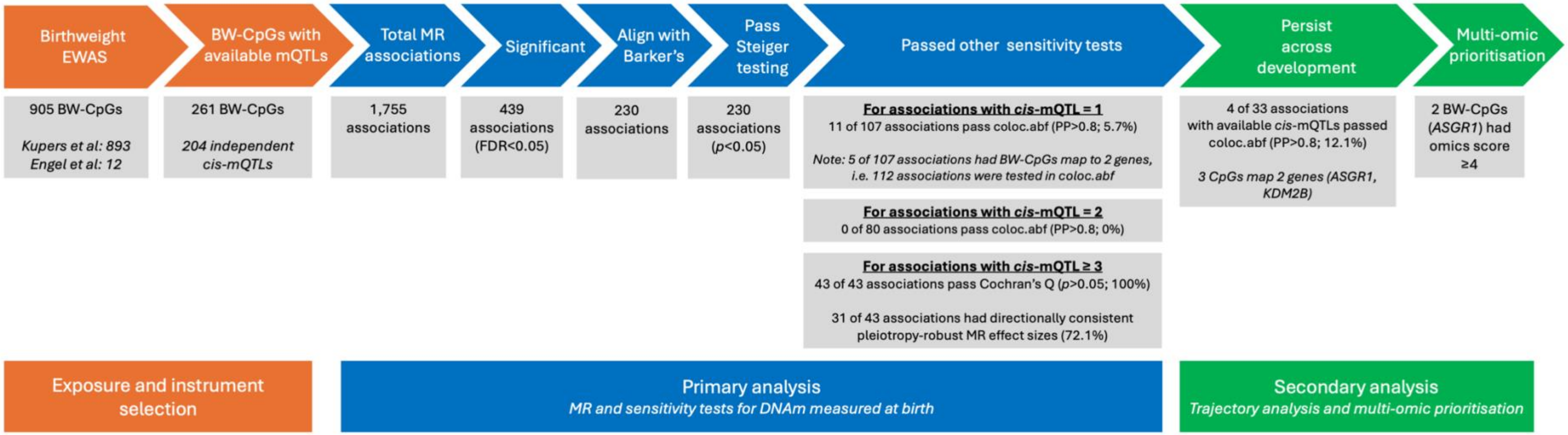

Figure S3. Flowchart of filtering steps and their results. Beginning with 905 BW-CpGs identified from two EWAS (Küpers et al: 893; Engel et al: 12), 261 BW-CpGs with 204 available and independent *cis*-mQTLs were carried forward. Epigenetic MR generated 1,755 total associations, of which 439 were significant (FDR<0.05), and 230 aligned with Barker's hypothesis and passed Steiger testing (p<0.05). Sensitivity analyses were stratified by the number of instruments: for single-instrument associations (*cis*-mQTL = 1), 11 of 107 passed colocalisation (PP>0.8; 5.7%; with a total of 112 colocalisation tests performed since 5 BW-CpGs mapped to two genes); for two-instrument associations (*cis*-mQTL=2), none of the 80 passed colocalisation; for associations with multiple instruments (cis-mQTL≥3), all 43 passed Cochran's Q (p>0.05; 100%), with 31 showing directionally consistent pleiotropy-robust effect sizes with the IVW method (72.1%). Trajectory analysis identified 4 of 33 associations with available *cis*-mQTLs that persisted across development that also passed colocalisation (12.1%). These associations involved 3 CpGs that mapped to *ASGR1* and *KDM2B*. Multi-omics prioritisation identified 2 BW-CpGs at *ASGR1* with an omics score greater than 4. BW-CpG,

birthweight-associated CpG site; DNAm, DNA methylation; EWAS, epigenome-wide association study; FDR, false discovery rate; MR, Mendelian randomisation; mQTL, methylation quantitative trait locus; PP, posterior probability.

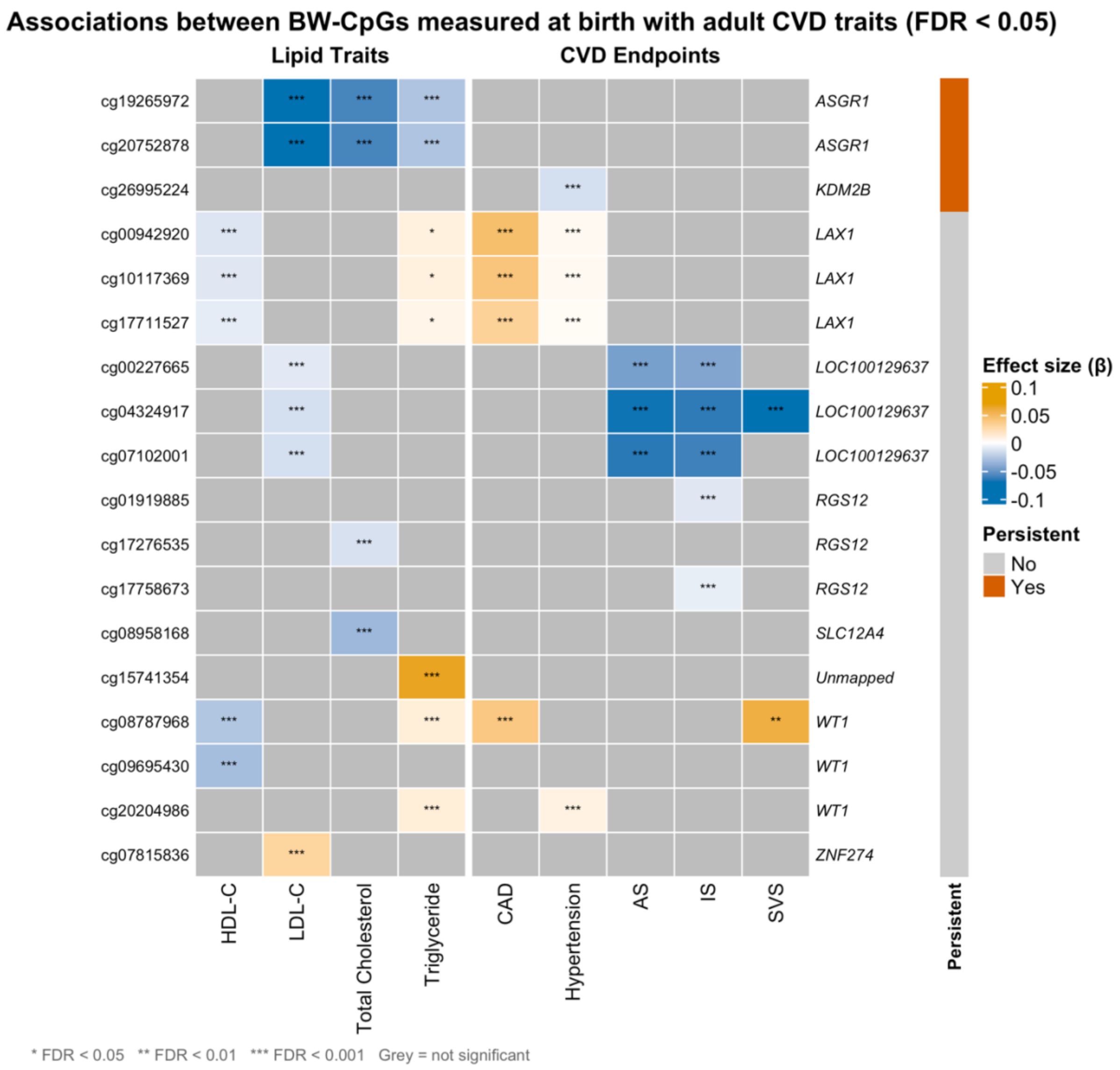

Figure S4. Heatmap of significant and robust MR associations between BW-CpGs and adult CVD traits, using mQTLs measured at birth, with persistent associations across development. Effect sizes (β) are shown for epigenetic MR analyses between BW-CpGs (left axis) and adult lipid traits and cardiovascular endpoints (bottom axis) that passed all sensitivity analyses. The colour intensity for effect size reflects the magnitude of the causal estimate, with blue indicating negative (protective effect) and orange indicating positive (increases risk) effect directions. Grey cells indicate non-significant associations. BW-CpGs are annotated by their nearest gene (right axis); those with persistent DNAm changes from birth through adolescence are highlighted in orange in the "Persistent" column. Significance thresholds: * FDR < 0.05;

** FDR < 0.01; *** FDR < 0.001. AS, any stroke; BW-CpG, birthweight-associated CpG site; CAD, coronary artery disease; CVD, cardiovascular disease; DNAm, DNA methylation; FDR, false discovery rate; HDL-C, high-density lipoprotein cholesterol; IS, ischaemic stroke; LDL-C, low-density lipoprotein cholesterol; MR, Mendelian randomisation; SVS, small-vessel stroke.
